# *Mycobacterium bovis* uses the ESX-1 Type VII secretion system to escape predation by the soil-dwelling amoeba *Dictyostelium discoideum*

**DOI:** 10.1038/s41396-019-0572-z

**Published:** 2020-01-02

**Authors:** Rachel E Butler, Alex A. Smith, Tom A. Mendum, Aneesh Chandran, Huihai Wu, Louise Lefrançois, Mark Chambers, Thierry Soldati, Graham R Stewart

**Affiliations:** 1 Department of Microbial Sciences, School of Biosciences and Medicine, University of Surrey, Guildford, Surrey GU2 7XH UK; 2 Department of Biochemistry, Science II, University of Geneva, 30 quai Ernest-Ansermet, Geneva, Switzerland; 3 School of Veterinary Medicine, University of Surrey, Guildford, Surrey GU2 7AL UK

**Keywords:** Bacterial genetics, Bacterial pathogenesis

## Abstract

*Mycobacterium bovis* is the causative agent of bovine tuberculosis and the predominant cause of zoonotic tuberculosis in people. Bovine tuberculosis occurs in farmed cattle but also in a variety of wild animals, which form a reservoir of infection. Although direct transmission of tuberculosis occurs between mammals, the low frequency of contact between different host species and abundant shedding of bacilli by infected animals suggests an infectious route via environmental contamination. Other intracellular pathogens that transmit via the environment deploy strategies to survive or exploit predation by environmental amoebae. To explore if *M. bovis* has this capability, we investigated its interactions with the soil and dung-dwelling amoeba, *Dictyostelium discoideum*. We demonstrated that *M. bovis* evades phagocytosis and destruction by *D. discoideum* and actively transits through the amoeba using the ESX-1 Type VII Secretion System as part of a programme of mechanisms, many of which have been co-opted as virulence factors in the mammalian host. This capacity of *M. bovis* to utilise an environmental stage between mammalian hosts may enhance its transmissibility. In addition, our data provide molecular evidence to support an evolutionary role for amoebae as training grounds for the pathogenic *M. tuberculosis* complex.

## Introduction

*Mycobacterium bovis*, a member of the *M. tuberculosis* complex, causes bovine tuberculosis, one of the most important veterinary health problems in the UK and Ireland [1, 2]. It is also a neglected zoonotic infection in humans, causing an estimated 147,000 new infections and 12,500 deaths worldwide in 2016 [3, 4]. In addition to farmed cattle, a wide range of wild mammals harbours the disease including badgers (UK), wild boar and red deer (continental Europe) and brushtail possums (New Zealand), and likely act as endemic wildlife reservoirs for the disease [5, 6]. Transmission from a wildlife reservoir in the UK and Ireland is thought to be a prominent factor undermining the effectiveness of control measures based on cattle test and slaughter. Transmission of bovine TB is primarily mediated through inhalation of aerosols from infected animals during close contact [7]. However, the exact means of transmission to and from the wildlife reservoir remains to be fully elucidated. For example, tracking of cattle and badgers has shown that they do not come into close contact on pasture [8]. However, badgers and cattle have been shown to secrete bacilli in their urine and/or faeces, and *M. bovis* has been detected in the soil and pasture near badger setts [9, 10]. This, and the finding that *M. bovis* can survive for extended periods in soil [11], has led researchers to postulate that indirect transmission through environmental contamination may facilitate the spread of bovine tuberculosis [1, 2, 12]. Although *M. tuberculosis* complex organisms are host-adapted species, their evolutionary origins lie with environmental organisms [13, 14]. As such, both *M. bovis* and its non-pathogenic progenitors are likely to have interacted with unicellular eukaryotes, such as species of amoeba that are abundant in terrestrial or aquatic environments.

It is postulated that contact with amoebae may have shaped the evolution of mycobacteria [15–18] with the amoeba intracellular environment acting as an evolutionary “training ground” for pathogenic bacteria [19–23]. Studies on *Mycobacterium marinum*, a pathogen of poikilotherms such as fish and frogs [14, 24–26] and the environmental opportunistic pathogens of the *M. avium* complex [27, 28], support this view, with overlap in the virulence mechanisms/genes used to replicate and survive in both amoebae and the phagocytes of higher organisms. In *M. marinum*, this includes the involvement of the ESX-1 Type VII Secretion System to effect phagosome escape [17, 29–31].

Given the potential importance of an environmental element to the transmission cycle of *M. bovis* between hosts, we surmised that it would be advantageous for *M. bovis* to possess active survival mechanisms to endure predation by environmental amoebae. To investigate this, we examined the uptake, survival and escape of this mammalian pathogen upon interaction with the free-living soil and dung-dwelling amoeba, *Dictyostelium discoideum* [32]. Further, we screened *M. bovis* genetic mutants to reveal that escape from the host amoeba required numerous genes/molecular mechanisms that are also required for full virulence of *M. tuberculosis* complex bacteria in mammals. This is supportive of an evolutionary history in which adaptation to intracellular survival first occurred in amoebae and was later co-opted by the *M. tuberculosis* complex and other pathogenic mycobacteria for infection of phagocytes from vertebrates.

## Materials and methods

### Bacterial strains and culture methods

*Mycobacterium bovis* AF2212/97 [33], Genbank Accession NC_00000962, was maintained on Middlebrook 7H11 solid medium containing 40 mM sodium pyruvate, 0.5% lysed defibrinated sheep blood, 5% heat inactivated foetal bovine serum and 10% oleic acid-albumin-dextrose-catalase (OADC) supplement. *M. bovis* was cultured as a liquid broth in 7H9 medium containing 40 mM sodium pyruvate, 0.05% Tween®80 and 10% OADC. *M. marinum* M strain, *M. bovis* BCG and *M. smegmatis* mc^2^155 were maintained on 7H11 solid medium supplemented with 0.5% glycerol and 10% OADC, and in liquid broths using 7H9 medium supplemented with 0.05% Tween®80, 0.2% glycerol and 10% OADC.

### Construction of a Δ*espAC* mutant strain of *M. bovis*

A double knockout *M. bovis* strain of *espA* (Mb3646c; Rv3616c) and *espC* (Mb3645c; Rv3615c) was made using plasmids and primers as previously reported [34]. Genomic sequence upstream (left flank) and downstream (right flank) of Mb3645c-3646c were PCR amplified and cloned either side of the zeocin antibiotic resistance cassette of the cosmid pANE001 (pYUB854) [35] with the hygromycin cassette replaced with a zeocin cassette. Transduction phage were constructed and transduced into *M. bovis* using the pHAE159 mycobacteriophage-based method of transduction [35], and recovered on 7H11 with 25 μg/ml zeocin. Knockouts were confirmed by PCR using primers outside of the upstream and downstream flanking regions both alone and in combination with antibiotic cassette specific primers.

### Culture/Infection of *D. discoideum* with bacteria

The axenic *D. discoideum* laboratory strain AX2 was maintained at 20–80% confluence in 10-cm tissue culture dishes (Falcon) in HL5c medium including glucose and supplemented with vitamins and microelements (Formedium, Hunstanton, UK). For maintenance, penicillin (100 U/ml) and streptomycin (100 µg/ml) were included in the culture medium. For cultures with bacteria, *D. discoideum* were seeded into dishes in medium without penicillin and streptomycin.

For bacterial survival assays in *D. discoideum*, *M. bovis, M. marinum and M. smegmatis* were prepared for infection by harvesting at mid-late log phase (OD 0.8–1.0), centrifuging at 3700 × *g* for 20 min and re-suspending in HL5c medium without antibiotics. Confluent monolayers of 2 × 10^7^ were gravity infected with an MOI of 10 bacilli per amoeba for 2.5 h. The supernatant was removed and the monolayer of amoebae was washed extensively with HL5c medium to remove extracellular bacilli. *D. discoideum* were harvested by gently scraping, diluted and seeded in tissue culture flasks. Where indicated, 5 μg/ml streptomycin was added to the culture medium to prevent extracellular proliferation. *D. discoideum* were harvested by gently scraping and by centrifugation at 1500 × *g* for 10 min. *D. discoideum* were lysed using 0.1% Triton X-100 in water, and the bacilli released were recovered onto 7H11 medium. For differential separation of *D. discoideum*-associated and extracellular bacilli, the *D. discoideum* fraction was separated by centrifugation at 250 × *g* for 10 min, before recovery of bacilli by centrifugation at 3700 × *g* for 30 min.

For bacterial uptake assays, *D. discoideum* were cultured with FITC-labelled K12 *E. coli* (Life Technologies, Carlsbad, CA, USA), and FITC-labelled *M. bovis* BCG [36] for 2 h before washing in HL5c medium to remove extracellular bacteria, gently scraping to a suspension, washing with Sorensen’s phosphate buffer containing 5 mM sodium azide, and re-suspending in Sorensen’s phosphate buffer containing 120 mM sorbitol. Phagocytosis was determined by flow cytometry using an Attune Flow Cytometer (Life Technologies, Carlsbad, CA, USA), using the gating strategy described by Hagedorn and Soldati [37]. To assess internalisation by confocal microscopy, *D. discoideum* were seeded in 35 mm glass-bottomed imaging dishes (ibidi, Graefelfing, Germany) and infected with FITC-labelled *M.* *bovis* BCG as before. External bacteria were gently rinsed away and the amoebae fixed for 3 h at 4 °C with 4% methanol-free paraformaldehyde in Sorensen’s phosphate buffer. Amoebae were rinsed with Sorensen’s phosphate buffer and permeablised for 3 min at room temperature with PBS containing 0.1% Triton X-100. Actin was stained with ActinRed555 ReadyProbes reagent and nuclei visualised with NucBlue Fixed Cell ReadyProbes reagent (both from Molecular Probes). Amoebae were rinsed again and mounted with a coverslip and Prolong Gold (Molecular Probes). Amoebae were visualised using a Nikon A1M Confocal Microscope, using a ×60 oil immersion lens, and sequential scanning using the 405 nm, 488 nm and 561 nm laser lines. For assessing the total DNA content of cultures, *M. bovis* was fixed for 24 h with 4% paraformaldehyde, washed with PBS, and DNA labelled with SYTOX Green nucleic acid stain (Molecular Probes). Fluorescence measurements were performed using an Ascent Fluorescent plate-reader in black walled clear bottomed plates using the FITC filter sets.

### *M.bovis* transposon mutant library construction and selection for mutants trapped in *D. discoideum*

A transposon mutant library of *M. bovis* AF2212/97 was generated according to Long et al. [38]. Briefly, 100 ml of late log *M. bovis* culture was washed twice in MP buffer (50 mM Tris-HCl, pH 7.5, 150 mM NaCl, 10 mM MgSO_4_ and 2 mM CaCl_2_) at 37 °C, and then incubated with more than 1 × 10^11^ pfu of φMycoMarT7 phage for 4.5 h. The suspension was recovered by centrifugation, washed and plated on ten 15-cm 7H11 plates supplemented with 20 μg/ml kanamycin and 0.02% Tween®80. After 18 days the library was scraped from the plates, dispersed in broth overnight and frozen in aliquots at −80 °C in 10% glycerol. For library selection, *M. bovis*-Tn library was thawed and washed in 7H9 before recovering in 7H9 complete medium containing 30 μg/ml kanamycin at 37 °C for 4 days. Mid log (OD 600 0.4–0.6) bacilli were prepared as before for infection, and used to infect 4 × 10^7^ *D. discoideum* at an MOI of 10 for 3 h in tissue culture coated dishes. In addition, 2 × 10^7^ cfu of library was plated onto 5 × 15 cm dishes of 7H11 containing 30 μg/ml kanamycin as the library reference control for each experiment. After 3 h, *D. discoideum* were washed with HL5c medium to remove extracellular bacilli, gently scraped and diluted into large tissue culture flasks. Streptomycin was omitted from these experiments as HL5c medium does not support the in vitro replication of *M. bovis* (Supplementary Fig. 1). After 2 days, *D. discoideum* were gently scraped and separated from extracellular bacteria by differential centrifugation. Bacilli were released from *D. discoideum* by lysing as before, and plating onto 5 × 15 cm dishes of 7H11 supplemented with 30 μg/ml kanamycin. After 3–4 weeks, recovered *M. bovis* was harvested and stored in 10% glycerol at −80 °C. Four independent co-culture/infections were performed, three of which included technical duplicates that were harvested and sequenced independently.

### TnSeq: Genomic DNA preparation and transposon-insertion site sequencing

*M. bovis* mutant pools were resuspended in TE buffer pH 8.0, and incubated for 5 min rocking with an equal volume of methanol:chloroform 2:1. Bacilli were centrifuged and the pellet dried, before re-suspending in phenol:chloroform:isoamyl alcohol 25:24:1. Cells were disrupted with the Fastprep homogeniser (MP Biomedicals, Irvine, CA, USA) and lysing matrix B, and centrifuged to separate the aqueous and organic phases. The upper aqueous phase was re-extracted with an equal volume of phenol:chloroform:isoamyl alcohol 25:24:1, followed by 2 further extractions with chloroform. gDNA was precipitated with 0.1 volume 3 M sodium acetate pH 5.2 and 1 volume propan-2-ol, pelleted by centrifugation, washed with 70% ethanol, and resuspended in TE. Transposon junctions were amplified essentially as described in [34]. Briefly, a 5 μg aliquot of DNA was sheared in a Covaris® Sono 7 machine (Covaris Inc, Woburn, MA, USA) and purified using SPRIselect beads (Beckman Coulter, High Wycombe, UK) at 1× concentration. The DNA fragment ends were repaired, blunt ended, and ‘A’ overhangs added, using the NEBNextEnd repair/dA-Tailing Module protocol (NEB, Hitchin, UK). Linkers (annealed Adap1 and Adap2, Supplementary Table 1) were ligated to the ‘A’ tail ends at ×100 molar excess with the NEBNext Ultra II Ligation module (NEB) and the fragments purified with SPRIselect as before. Transposon junctions were amplified using primers IS6 and MarA to MarO (Supplementary Table 1), cycle conditions were 95 °C for 10 s, 58 °C for 10 s, 72 °C for 30 s. The primers have a P5-index that identifies the sample and a random P7-index to allow PCR-generated artefacts to be identified and removed from the data. Real-time PCR was used to determine the minimum number of cycles required to amplify the transposon junctions. PCR products were purified with SPRIselect and equimolar quantities of each preparation sequenced using a HiSeq® 2500 (Illumina, San Diego, CA, USA) with a single sequence read and double index reads.

### Analysis of transposon site data

Sequence data were demultiplexed using the P5-index reads, and quality controlled and aligned as described [39]. An additional step [34] removed artefactual amplicons generated by the PCR amplifications, which were identified as those reads having both identical P7-indexed reads and the same insertion site. Reads were aligned to the *M. bovis* AF2212/97 genome (NC_00000962) using Bowtie and the transposon counts determined. Gene essentiality in the input library was predicted using the TRANSIT Hidden Markov model (HMM) method [40] but excluding TA sites with a non-permissive motif as identified previously in *M. tuberculosis* [41]. We determined the mutants that were significantly enriched in the intracellular Dictyostelium fraction by comparing mutant abundance (numbers of each unique TnSeq read per gene) in the Dictyostelium-trapped fraction and the inoculating mutant library using the resampling option in the TRANSIT software with nzMean normalisations and non-permissive sites removed. This test uses a variation of the classical permutation statistical test, to provide a *p* value, which is adjusted for multiple comparisons by the Benjamini Hochberg procedure to provide a *q* value. Mutants were considered to be enriched or depleted in a condition at *q* < 0.05. The significance of gene group/pathway enrichments were determined using Fisher’s exact test

## Results

### *Mycobacterium bovis* survives predation by *Dictyostelium discoideum* amoeba

**Fig. 1:**
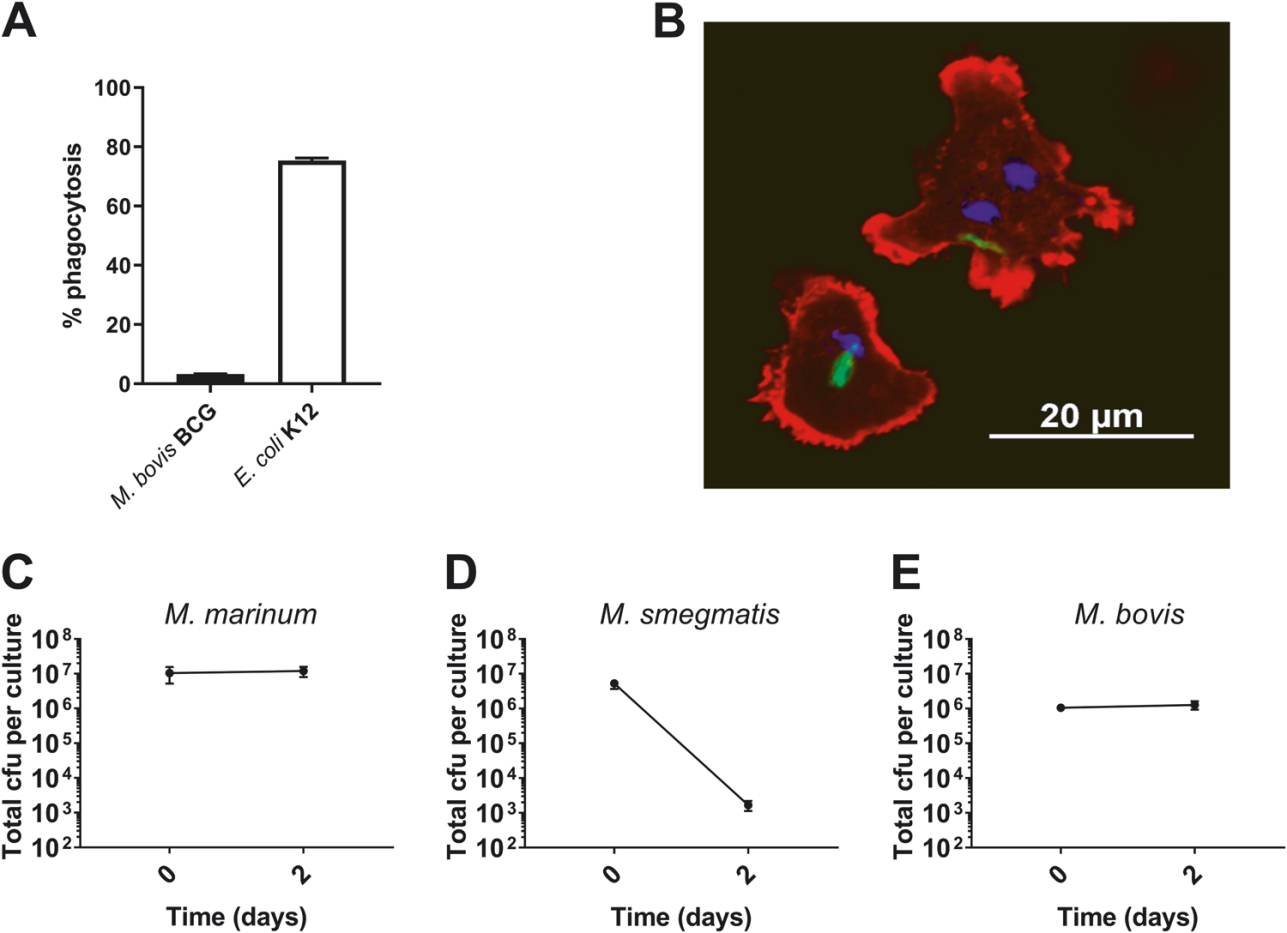
*Mycobacterium bovis* resists phagocytosis and killing by *D. discoideum.* **a** The percentage of *D. discoideum* that have phagocytosed *E. coli* K12-FITC or *M. bovis* BCG-FITC after a 2 h incubation at an MOI of 10 was determined by flow cytometry. The mean of three biological replicates ± SEM is displayed. **b** Representative micrograph of *D. discoideum* demonstrating internalised *M. bovis* BCG-FITC (green—*M. bovis* BCG-FITC; red—actin; blue—nuclei). *D. discoideum* was infected with an MOI of 10 with (**c**) *M. marinum* M strain, (**d**) *M. smegmatis* mc^2^155 strain, or (**e**) *M. bovis* AF2122/97. Cfu of bacteria per culture of 2 × 10^7^ amoebae was enumerated at the point of infection and after 2 days to determine bacterial survival. Streptomycin was included in the medium post infection to inhibit extracellular replication of bacteria. The mean and SEM of *n* = 3 independent experiments is displayed.

*D. discoideum* is a bacterivorous amoeba that survives in soil by preying on bacteria. To assess whether *M. bovis* could be a predated food source for *D. discoideum*, we compared the efficiency of uptake of FITC-labelled *M. bovis* BCG and *E. coli* K12 by flow cytometry. As seen in Fig. 1a, *M. bovis* BCG is poorly phagocytosed by *D. discoideum* in comparison with *E. coli* K12. However, internalisation of *M. bovis* BCG was confirmed by confocal microscopy (Fig. 1b). We next investigated the survival of *M. bovis* AF2122/97 in *D. discoideum* over 2 days of infection, and compared it with the survival of the pathogenic mycobacterial species *M. marinum* and non-pathogenic *M. smegmatis*. As seen in Fig. 1c–e, and in agreement with Hagedorn et al. [37], the pathogenic mycobacterial species *M. marinum* survives a 2-day infection in *D. discoideum*, whereas non-pathogenic *M. smegmatis* is killed by amoeba. We demonstrate that *M. bovis* also survives for 2 days in *D. discoideum*. Taken together, our data demonstrate that *M. bovis* is inefficiently phagocytosed as prey by *D. discoideum*; however, once phagocytosed, *M. bovis* is resistant to intracellular killing.

### *Mycobacterium bovis* replicates at ambient temperature

**Fig. 2:**
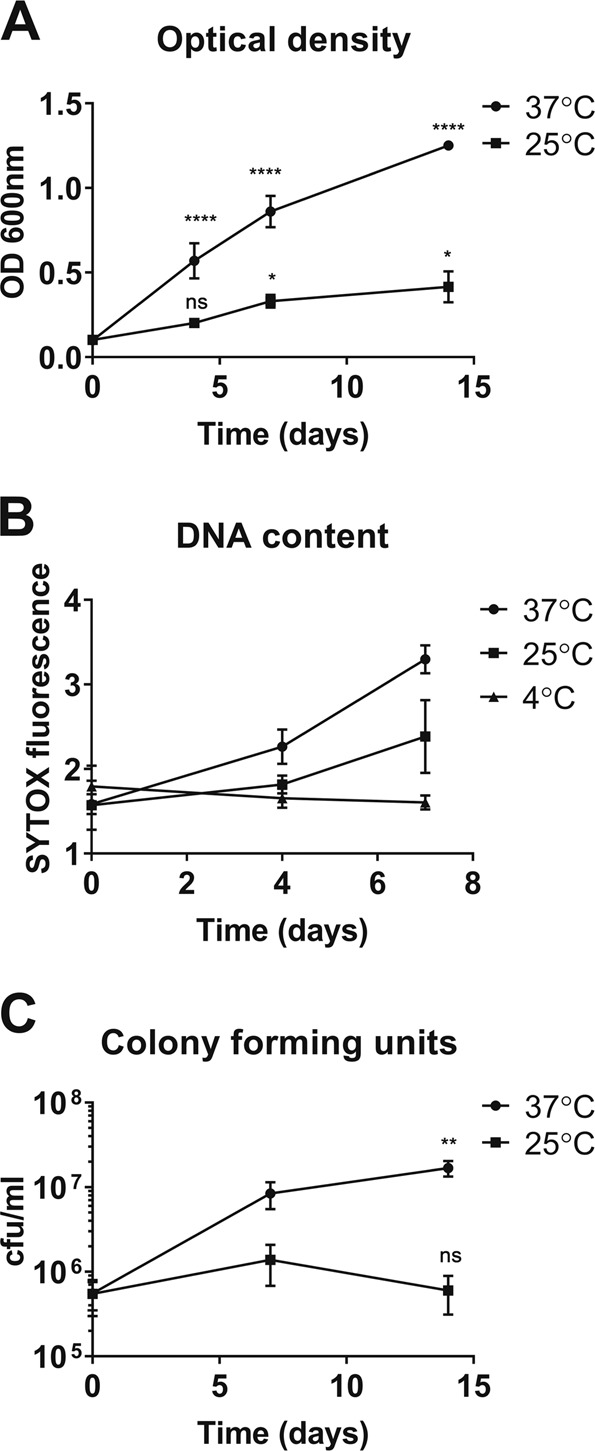
*Mycobacterium bovis* slowly replicates for up to 7 days at 25 °C. **a** Growth of *M. bovis* AF2122/97 was compared at 25 and 37 °C by optical density (OD600 nm) at 0 (*n* = 4), 4 (*n* = 3), 7 (*n* = 4) and 14 (*n* = 2) days. The mean and SEM of independent experiments (each performed in biological duplicate or triplicate) is displayed. Growth was compared with day 0 by two-way ANOVA (*p < 0.05; **p < 0.01; ***p < 0.001; ****p < 0.0001). **b** DNA synthesis in cultures was measured using Sytox Green fluorescent dye, (mean of three biological replicates ± SEM is displayed). **c** Growth of *M. bovis* AF2122/97 was compared at 25 and 37 °C by counting colony forming units. The mean and SEM of *n* = 2 independent experiments (each performed in biological triplicate) is displayed. Growth was compared with day 0 by two-way ANOVA (**p < 0.01).

*D. discoideum* is unable to tolerate the high temperatures of warm-blooded animals, with an upper temperature growth limit of around 25.5 °C. The environmental species *M. marinum* has an overlapping temperature range and thus is able to divide in *D. discoideum* at 25 °C. However, as a pathogen of mammals, *M. bovis* replicates in cattle with a body temperature of 38.5 °C, and as such might not be expected to efficiently replicate at lower temperatures. We compared the growth of *M. bovis* in optimal growth medium at 25 and 37 °C. As seen in Fig. 2, over 14 days of growth the optical density of the cultures increases, indicating slow but detectable bacterial division at 25 °C (Fig. 2a). By using a dye to measure total DNA content in the culture, we confirmed that the increase in culture density was indeed due to bacterial division rather than an artefact caused by the disaggregation of bacterial clumps (Fig. 2b). Counting cfu revealed that there was an apparent balance of division and progression to a non-culturable state in the 25 °C cultures, such that the viable plate count did not significantly increase over the 14-day time period analysed (Fig. 2c). Taken together, our data demonstrate that *M. bovis* is metabolically active and divides at a much lower temperature than previously reported. This observation is important because it allows for the possibility that *M. bovis* survival in *D. discoideum* is an active process. Furthermore, survival/adaptation to ambient temperatures may partially explain the success of *M. bovis* transmission between cattle, and between cattle and wildlife reservoirs, which has been postulated to occur via an intermediate environmental step.

### *Mycobacterium bovis* uses the ESX-1 secretion system to escape from *D. discoideum*

**Fig. 3:**
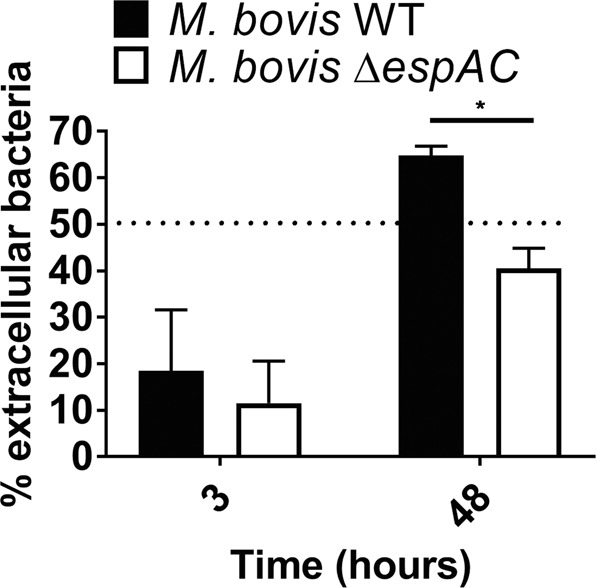
*M. bovis* Δ*espAC* mutants defective in the ESX-1 machinery have impaired ability to escape from *D. discoideum.* *D. discoideum* were infected at MOI 10 with *M. bovis* wild-type (WT) and *M. bovis* Δ*espAC*. After 3 h and 48 h, *D. discoideum* and extracellular bacteria were separated by differential centrifugation, and bacilli enumerated after recovery on solid medium. The experiments were performed in the absence of streptomycin; the HL5c growth medium does not support the extracellular replication of *M. bovis* (Fig. S2). The mean and SEM of *n* = 2 independent experiments (each performed in biological duplicate or triplicate) is displayed. Statistical analysis was performed by Student’s *t* test (*p < 0.05).

The ESX-1 locus encodes a Type VII Secretion System that is required for virulence in *M. bovis* and *M. tuberculosis* in mammals [30, 42–45]. Mycobacteria with mutations in the ESX-1 machinery have been shown to have reduced fitness in mammalian phagocytes, mice and guinea pigs [39, 46–48]. The ESX-1 machinery transports virulence factors into host cells, including the immunodominant co-secreted effectors, EsxA (ESAT-6) and EsxB (CFP-10). EsxA has the ability to disrupt cell membranes [49, 50], potentially catalysing the ESX-1-dependent exit of *M. tuberculosis* complex bacteria from the phagosome of mammalian cells into the cytosol [51–55] and ultimately inducing host cell lysis and bacterial release [50, 56–59]. To determine if ESX-1 plays a role in *M. bovis* survival in amoebae, we compared the ability of wild type and ESX-1 defective *M. bovis* to transit through *D. discoideum*. We constructed a mutant of the *espACD* operon, which is essential for ESX-1-dependent EsxA/EsxB secretion and pathogenic function [60–62]. *D. discoideum* were allowed to ingest wild-type *M. bovis* and *M. bovis* Δ*espAC*, and, after 48 h, Dictyostelium-associated bacilli and extracellular bacilli were separated by differential centrifugation. As seen in Fig. 3, over 60% of wild-type *M. bovis* escaped from *D. discoideum* but loss of ESX-1 function by deletion of *espAC* impaired *M. bovis* escape. This contrasts with *M. marinum* where the loss of ESX-1 alters the route of bacterial release, reducing cytolysis but actually increasing transit [63]. There was no difference in the cytotoxic capacity of *M.bovis* wild-type and Δ*espAC* strains (Supplementary Fig. 2). In conclusion, *M. bovis* actively transits though *D. discoideum*, in part using the ESX-1 locus.

### *M. bovis* genes associated with virulence in vertebrates are required for escape from *D. discoideum*

**Fig. 4:**
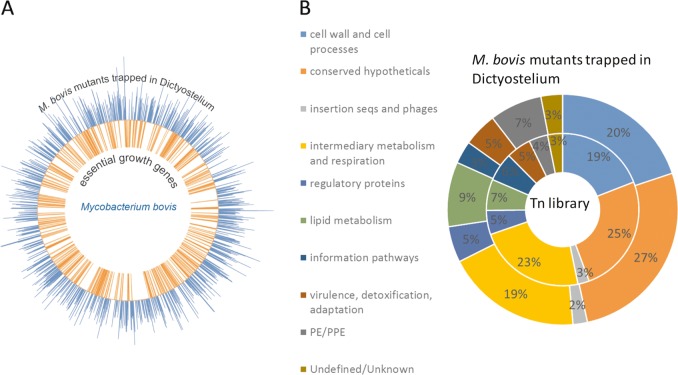
Distribution of transposon-insertion sites in the *Dictyostelium discoideum*-trapped *Mycobacterium bovis* mutant pool. **a** The distribution of transposon-insertion sites around the bacterial chromosome that result in defective transit through *D. discoideum* are depicted in the outer ring (in blue). The line length represents the fold change after 2-day infection in *D. discoideum*, compared with the inoculum. The distribution of essential growth genes is represented by the inner ring (rust). **b** The distribution of mutations associated with defective transit through *D. discoideum* is displayed by functional class (outer ring). The distribution of genes containing transposon insertions in the unselected inoculum library is represented by the inner circle.

To further understand the involvement of ESX-1 in *M. bovis* escape from *D. discoideum* and to investigate other genes/systems involved in this interaction, we performed a genome-wide screen of *M. bovis* transposon-insertion mutants, selecting for those which had an impaired ability to transit through *D. discoideum*. A pool of ~3.5 × 10^5^ individual mutants was generated and sequencing revealed that it contained ~43,000 unique transposon-insertion sites and 318 genes were categorised to be essential by analysis with TRANSIT HMM (Fig. 4a, Supplementary Table 2). *D. discoideum* was infected with the mutant library, and after 48 h the transposon mutants that remained trapped inside the amoebae were recovered by differential centrifugation and lysis of *D. discoideum*. This provided a *positive* selection for mutants with defective transit through the amoebae. *M. bovis* mutants from the *D. discoideum*-associated fractions of four independent experimental infections were recovered on 7H11 medium and the relative abundance of mutants in each pool compared by TnSeq with similarly recovered mutant pools of the inocula [40]. Mutations in 694 genes were significantly enriched in the intracellular *D. discoideum* fraction (Supplementary Table 2) indicative of defective transit through *D. discoideum*.

Genes required for transit through *D. discoideum* were distributed widely throughout the mycobacterial chromosome, including in the chromosomal regions that are overtly devoid of essential growth genes (Fig. 4a). We analysed the distribution of these mutations within functional categories of genes assigned by Mycobrowser (Fig. 4b). Mutations associated with impaired escape from *D. discoideum* are distributed throughout all functional classes, reminiscent of virulence gene datasets for *M.tuberculosis* in vertebrates [47, 64]. We further analysed the amoeba-trapped mutations to assess the statistical significance of the “escape” phenotype with known gene families, pathways and functional groups in *M. bovis* (Table 1).

**Table 1:** Functional group analysis of mutants attenuated for *Dictyostelium discoideum* escape.

Gene locus/Functional group/family	Enrichment in Dictyostelium (significance *p* < 0.05)
Type VII secretion systems	
ESX-1	*p* = 0.0003
ESX-2	ns
ESX-3	Essential system
ESX-4	ns
ESX-5	In vitro growth defect
PE/PPE	
PE	*p* = 0.0145
• PE-only	ns
• PE_PGRS	*p* = 0.0081
PPE	*p* = 0.0002
MCE transporters	
MCE1	ns
MCE2	ns
MCE4	*p* = 0.0202
Other	
Serine/Threonine protein kinases	*p* = 0.0465
Sulfolipid synthesis/Transport	*p* = 0.0110
LipC-LipZ lipases	*p* = 0.0131

Gene loci/Families/Functional groups/Pathways were tested for significance using resampling analysis in TRANSIT and Fisher’s exact test

#### ESX-1 secretion system

**Fig. 5:**
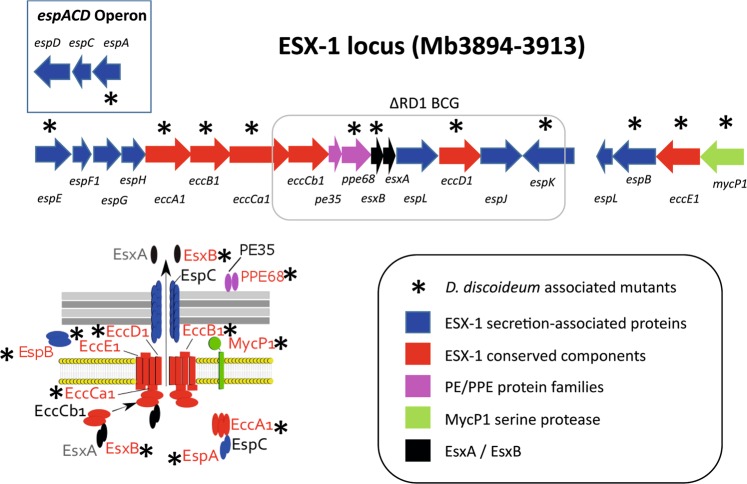
ESX-1 mutations (asterisks) that attenuate *Mycobacterium bovis* escape from *Dictyostelium discoideum.* A model for ESX-1 secretion (adapted from [89–91]). EsxA/EsxB and EspA/EspC bind to cytoplasmic ATPases, such as EccA1 and EccCb1, via the C-terminals of EsxB and EspC, respectively. The protein complex interacts with the translocation complex likely formed by the transmembrane proteins EccCa1, EccB1, EccD1, EccE1 and MycP1 [90]. When uncoupled from the chaperone protein EspA, EspC is able to polymerise into filaments which may form the secretion system across the mycomembrane [92].

Consistent with findings that the ESX-1 machinery is required for transit of *M. bovis* through *D. discoideum*, we found that mutations in 12 genes of the extended ESX-1 locus (ESX-1 Mb3894-3913, plus the *espACD* operon and EspR regulator) are enriched in the intracellular *D. discoideum* mutant pool (Supplementary Table 2, Table 1 and Fig. 5). By contrast, mutations in the related type VII secretion systems ESX-2, ESX-3, ESX-4 and ESX-5 were not significantly associated with impaired transit through amoebae. However, as ESX-3 is an essential system and mutations in ESX-5 cause an in vitro growth defect (Supplementary Fig. 3), definitive conclusions about their specific involvement in the bacterium-amoeba interaction cannot be drawn.

#### PE/PPE proteins

The PE/PPE proteins are unique to mycobacteria and have been previously associated with virulence in vertebrates. Here, mutants carrying insertions in PE/PPE family proteins were prominently enriched in *D. discoideum* (Fig. 4b, dark grey segments, Table 1) with mutation of 27 PE and 24 PPE genes conferring attenuation for amoeba transit (Supplementary Table 2). Further analysis of the PE family revealed that the mutants of 20 PE-PGRS proteins were defective for amoeba escape.

#### MCE4 lipid transporter and lipases

Mutants in genes encoding the virulence-associated MCE4 cholesterol transport system [65] were also indicated as trapped in *D. discoideum* infection (Table 1, Supplementary Table 2, Supplementary Fig. 4). Export of the MCE4 machinery to the cell envelope has recently been shown to require the function of the SecA2 secretion apparatus [66]; we also find that the mutations in SecA2 are significantly enriched in our *D. discoideum* screen (Supplementary Table 2). Interestingly, mutations in Lip-family lipases (24 lipid/ester hydrolases called LipC to LipZ) were additionally found to be enriched in *D. discoideum* (Table 1, Supplementary Table 2), suggesting that utilisation of triacylglycerol-derived carbon may be required for optimal escape from amoeba.

#### Cell wall lipid synthesis

*M. tuberculosis* complex mutants lacking cell wall phthiocerol dimycocerosates (PDIM) are heavily attenuated in mammalian phagocytes [67] and act in concert with EsxA to disrupt phagosomal membranes [49, 68, 69] suggesting a mechanism by which the regulation of PDIM content of the cell envelope could aid escape from amoebae. However, although insertional mutations in the Mmpl7 PDIM transporter, and the key PDIM biosynthetic enzyme FadD26 [70] were found to be significantly enriched in *D. discoideum* (Supplementary Table 2), as a collective, insertional mutations in PDIM synthetic pathways were not significantly enriched. By contrast, mutations in genes encoding for the synthesis and transport of sulfolipid-1 (SL-1), a virulence-associated outer membrane glycolipid [71] were attenuated for *D. discoideum* escape (Table 1, Supplementary Table 2, Supplementary Fig. 4).

#### Other vertebrate-virulence/Dictyostelium-escape genes

Other parallels with *M. tuberculosis* virulence genes that may shed some light on the dynamic nature of the *D. discoideum*/*M. bovis* interaction are the importance of genes involved in the oxidative stress response *(oxyS* ROS-responsive regulator and the *katG* catalase gene) and genes for uptake of aspartate, *ansP1*, and asparagine, *ansP2*. [72] (Supplementary Table 2). Evidence that *M. bovis* needs to actively sense the intra-amoebic environment in order to respond and exit the amoeba host cell is supported by attenuation of a significant number of mutants in serine/threonine protein kinases (STPKs, Table 1 and Supplementary Table 2).

## Discussion

This study demonstrates for the first time that a member of the *M. tuberculosis* complex is adapted to actively combat predation/phagocytosis by amoebae at environmental temperature. This observation is important at two levels: understanding the transmission of *M. bovis* between animal hosts; and, understanding the evolution of pathogenesis in the *M. tuberculosis* complex. Whereas human tuberculosis is spread by aerosol infection directly between hosts in close contact, the transmission route of bovine tuberculosis between cattle and badgers remains to be fully elucidated. The secretion of *M. bovis* in urine and faeces of infected animals and the finding that *M. bovis* can survive for extended periods in soil, has led researchers to postulate that environmental contamination may facilitate the spread of bovine tuberculosis [1]. For this transmission route to be effective, we hypothesised that *M. bovis* possessed adaptations for environmental survival including mechanisms that allow it to survive predation by bacterivorous amoebae that reside in soil and dung [32], like *D. discoideum*.

Arguably the most significant observation of the present study was that *M. bovis* displayed active metabolism and cell division at 25 °C, thus it has the physiological opportunity to adapt to the environment, including amoebae, between vertebrate hosts. The role of amoebae in the transmission cycle of *M. bovis* has been a source of debate. One school of thought is that amoebae may provide a protective or replicative niche for the mycobacterium. Indeed, *M. bovis* has been shown to survive within the trophozoites of *Acanthamoeba polyphaga* and *A. castellanii*, [18, 73, 74] where a proportion become encased in a protective cyst and maintain the ability to cause infection in mice [73]. However, the majority of *M. bovis* bacilli bypass the amoeba cyst stage, possibly by exiting the protozoan via exocytosis or non-lytic ejection described in *D. discoideu*m [17, 18, 74]. Consistent with this, and coherent with our hypothesis, we show that the bacterium is not phagocytosed in large quantities by *D. discoideum* but when it is internalised it uses mechanisms encoded by a large set of genes to effect escape from the amoeba.

Intriguingly, many of the factors required for escape from *D. discoideum* are previously documented as virulence factors in mammalian hosts, most conspicuously the ESX-1 system. ESX-1 in *M. marinum* is crucial for parasitism of amoebae, mammalian phagocytes, and zebrafish models of infection [17, 29, 31]. However, our demonstration that the mammalian pathogen *M. bovis* also uses ESX-1 to evade the microbicidal mechanisms of amoebae establishes more firmly that amoebae could have acted as evolutionary “training grounds” for the ancestors of today’s *M. tuberculosis* complex. Supporting this conclusion is the importance of PE/PPE proteins to both the escape of *M. bovis* from amoebae and in subversion of vertebrate immunity and control of macrophage function [75, 76]. PE/PPE protein families are unique to mycobacteria, and their expansion in the *M. tuberculosis* complex, where they account for ~10% of the coding capacity of the genome [77] has been previously linked to adaptation to human and animal hosts [71, 78]. This is clearly illustrated in the most recently evolved group of PE proteins, the PE-PGRS sublineage, which are exclusively associated with the *M. tuberculosis* complex and related pathogenic mycobacteria such as *M. marinum* [76, 78]. The present study identified 20 PE-PGRS genes important for amoeba escape, twelve of which were previously identified as important for *M. tuberculosis* survival in human dendritic cells [39]. This is of particular interest given that PE-PGRS are not secreted in *M. bovis* due to RD5-deletion of *ppe38*, suggesting that the virulence phenotype of these genes is not dependent on secretion [79]. It is tempting to speculate that the genomic expansion of the extensive PE/PPE gene/protein families may have been driven by selective pressure from amoebae and the adaptation co-opted for survival in vertebrate phagocytes.

We have identified other known “mammalian virulence” gene families involved in escape from *D. discoideum*, including those encoding the MCE4 cholesterol transport pathway. MCE4 has been implicated in transport of lipids across the mycobacterial cell wall including uptake of cholesterol [65] and it is important for the maintenance of infection in the murine model of TB [47, 80]. Although *D. discoideum* does not synthesise cholesterol, instead synthesising sitosterol [81], our results are consistent with those in *M. marinum* where MCE4 orthologues are required for growth in *D. discoideum* suggesting a dietary switch to sterols as a conserved adaptation for intracellular mycobacteria in phagocytes [31]. We also identify cell wall SL-1, to be required for *D. discoideum* phagosome escape. SL-1 is a glycoprotein which is only found in pathogenic mycobacteria [82], and has previously been implicated in survival in human monocyte-derived dendritic cells [39]. This is particularly intriguing because *M. bovis* possesses only small amounts of SL-1 sulfolipid in its cell envelope compared with *M. tuberculosis* [83], due to mutation of the PhoP/R two-component regulatory system [84], and had thus been previously overlooked as a molecule of importance to *M. bovis* biology.

We found that mutants in several serine threonine protein kinases including PknH were impaired in their ability to escape from *D. discoideum* (Table 1 and S1). PknH phosphorylates the regulator protein EmbR in *M. tuberculosis*, which regulates arabinogalactan synthesis via the *embABC* genes, and may control the balance of lipoarabinomannan and lipomannan in the cell envelope [85]. *M. bovis* contains a second regulator EmbR2, which is thought to negatively regulate EmbR [86]. Mutations in both EmbR and Embr2 were also found to be enriched in *D. discoideum*. Collectively this suggests that regulating the arabinogalactan content of the cell wall may be required for optimal escape from amoeba. Escape defective *M.bovis* mutants also included those deficient in previously described virulence determinants involved in resistance to oxidative stress and nutrient acquisition. Thus, there appears to be considerable depth in the intracellular “virulence” adaptations co-opted between amoebae and vertebrate phagocytes.

One potential limitation of the assay is that mutants with profound intra-dictyostelium mortality may not be identified by the positive selection strategy for trapped-intracellular bacteria. However, our preliminary experiments with *M. bovis* Δ*espAC* suggested that transit-defective bacteria are not necessarily killed during the relatively short 48 h co-culture experiment. Thus we are confident that the vast majority of mutants with an altered intra-amoeba phenotype will be identified by the experiment.

We hope that our observations on *D. discoideum*/*M. bovis* interactions will encourage researchers to utilise *D.discoideum* as a useful, non-sentient, genetically tractable model of tuberculosis with the potential to reveal aspects of mycobacterial pathogenesis from an alternative but biologically relevant perspective. We recognise that there remains much to do to better characterise the system and develop longer term co-culture systems where the survival of bacterial mutation can be explored. Mycobacteria can exit amoebae via a range of mechanisms including exocytosis, direct lysis, and non-lytic ejection [17, 87, 88]. Further work will characterise the mechanisms of amoeba exit used by wild-type *M. bovis*, and how the gene families we have identified influence each of these.

In summary, we have demonstrated that *M. bovis* is resistant to uptake and destruction by environmental *D. discoideum* and actively transits through the amoeba using a programme of mechanisms, many of which have been co-opted as virulence factors in the mammalian host. This, combined with the ability to replicate and adapt at ambient temperatures, may enhance the capacity of *M. bovis* to utilise an environmental stage between mammalian hosts and so enhance transmissibility. Understanding the transmission of *M. bovis* is crucial for implementing effective control measures, and understanding the survival of *M. bovis* outside of mammalian hosts warrants further investigation.

## Supplementary information

Supplementary Table 1

Supplemmental Material

Supplementary Table 2

## Data Availability

The HiSeq datasets are available in the Sequence Read Archive (SRA) repository, Bioproject accession number PRJNA588141.
