# Supplementary material for: Mycobacterium bovis uses the ESX-1 Type VII secretion system to escape predation by the soil-dwelling amoeba Dictyostelium discoideum
Source: ISME J. 2020 Jan 2;14(4):919–30. doi: 10.1038/s41396-019-0572-z (PMC7082363; doi:10.1038/s41396-019-0572-z)
Supplement: Supplementary file 1 — Supplementary Table 1 [file 41396_2019_572_MOESM1_ESM.docx]

| Adap1 | **caagcAGAAGACGGCATACGAGAT**NNNNNNNNGTGACTGGAGTTCAGACGTGTGCTCTTCCgatct |
| --- | --- |
| Adap2 | gatcgGAAgagca-PHO |
| MarA | **AATGATACGGCGACCACCGAGATCTACACTGTTCCGA**ACACTCTTTCCCTACACGACGCTCTTCCGATCT**CGGGGACTTATCAGCCAACC** |
| MarB | **AATGATACGGCGACCACCGAGATCTACACTTCCGGAG**ACACTCTTTCCCTACACGACGCTCTTCCGATCT**TCGGGGACTTATCAGCCAACC** |
| MarC | **AATGATACGGCGACCACCGAGATCTACACGCCGATGT**ACACTCTTTCCCTACACGACGCTCTTCCGATCT**GATACGGGGACTTATCAGCCAACC** |
| MarD | **AATGATACGGCGACCACCGAGATCTACACCATGATCG**ACACTCTTTCCCTACACGACGCTCTTCCGATCT**TATCTACGGGGACTTATCAGCCAACC** |
| MarE | **AATGATACGGCGACCACCGAGATCTACACCGCGCGGT**ACACTCTTTCCCTACACGACGCTCTTCCGATCT**CGGGGACTTATCAGCCAACC** |
| MarF | **AATGATACGGCGACCACCGAGATCTACACACACGATC**ACACTCTTTCCCTACACGACGCTCTTCCGATCT**TCGGGGACTTATCAGCCAACC** |
| MarG | **AATGATACGGCGACCACCGAGATCTACACAAGTAGAG**ACACTCTTTCCCTACACGACGCTCTTCCGATCT**GATACGGGGACTTATCAGCCAACC** |
| MarH | **AATGATACGGCGACCACCGAGATCTACACACTTGA**ACACTCTTTCCCTACACGACGCTCTTCCGATCT**TATCT**A**CGGGGACTTATCAGCCAACC** |
| MarI | **AATGATACGGCGACCACCGAGATCTACACGATCAG**ACACTCTTTCCCTACACGACGCTCTTCCGATCT**CGGGGACTTATCAGCCAACC** |
| MarJ | **AATGATACGGCGACCACCGAGATCTACACTAGCTT**ACACTCTTTCCCTACACGACGCTCTTCCGATCT**TCGGGGACTTATCAGCCAACC** |
| MarK | **AATGATACGGCGACCACCGAGATCTACACGGCTAC**ACACTCTTTCCCTACACGACGCTCTTCCGATCT**GATACGGGGACTTATCAGCCAACC** |
| MarL | **AATGATACGGCGACCACCGAGATCTACACCTTGTA**ACACTCTTTCCCTACACGACGCTCTTCCGATCT**TATCTACGGGGACTTATCAGCCAACC** |
| MarM | **AATGATACGGCGACCACCGAGATCTACACAGTCAA**ACACTCTTTCCCTACACGACGCTCTTCCGATCT**CGGGGACTTATCAGCCAACC** |
| MarN | **AATGATACGGCGACCACCGAGATCTACACAGTTCC**ACACTCTTTCCCTACACGACGCTCTTCCGATCT**TCGGGGACTTATCAGCCAACC** |
| MarO | **AATGATACGGCGACCACCGAGATCTACACATGTCA**ACACTCTTTCCCTACACGACGCTCTTCCGATCT**GATACGGGGACTTATCAGCCAACC** |
| IS6 | **CAAGCAGAAGACGGCATACGA** |

Supplementary Table 1

Oligonucleotides, linkers and primers for TnSeq
