# Supplementary material for: Mycobacterium bovis uses the ESX-1 Type VII secretion system to escape predation by the soil-dwelling amoeba Dictyostelium discoideum
Source: ISME J. 2020 Jan 2;14(4):919–30. doi: 10.1038/s41396-019-0572-z (PMC7082363; doi:10.1038/s41396-019-0572-z)
Supplement: Supplementary file 2 — Supplemmental Material [file 41396_2019_572_MOESM2_ESM.docx]

Supplementary Data

**Figure S1. HL5c medium does not support the in vitro growth of *M. bovis*.** Growth of *M. bovis*, *M. bovis* BCG and the *M. bovis* transposon library (Tn) at 37°C for 3-4 days was compared in 7H9 medium supplemented with sodium pyruvate, OADC and Tween-80, and HL5c medium. Results are mean +/- SEM from 3 biological triplicates.

**Figure S2. Cytotoxicity of *M. bovis* to *D. discoideum* is not impaired by deletion of Δ*espAC*.** *D. discoideum* monolayers in tissue culture dishes were exposed to MOIs 0-50 of *M. bovis* AF2212/97 wild type (WT) and the ESX-1 defective mutant *M. bovis* Δ*espAC*. After 24 hours, the monolayers were fixed and attached cells measured using crystal violet staining. No difference in cytotoxicity was found between the two strains. Results are mean +/- SEM from biological triplicates.


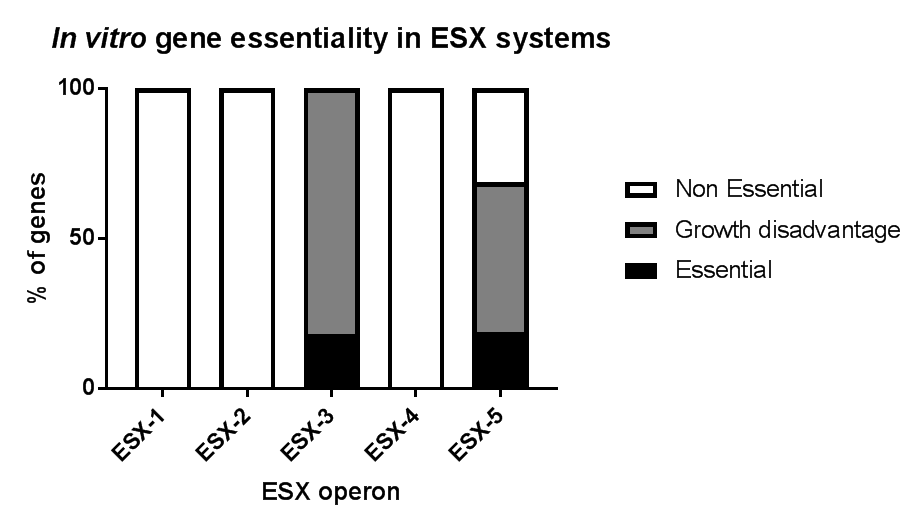


**Figure S3. In vitro gene essentiality in ESX operons in the *M. bovis* Tn-library.** Essentiality calls in the *M. bovis* transposon library were predicted by the TRANSIT HMM Analysis package. No transposon insertions were found to be growth advantageous in ESX1-5.


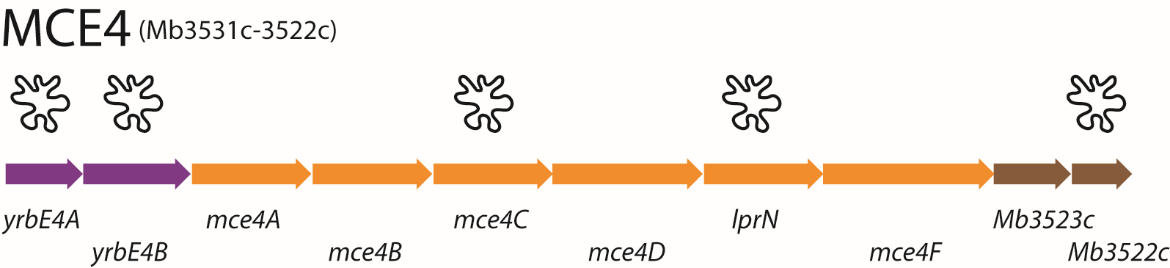


**
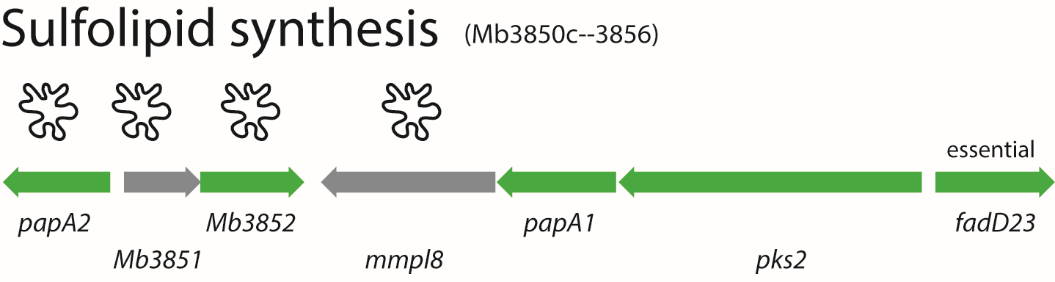
**

**Figure S4. MCE4 and sulfolipid synthesis are required for *D. discoideum* escape by *M. bovis*.** Mutants trapped in *D. discoideum* are indicated by amoeba cartoons.
